# A naturally-occurring ‘cold earth’ spot in Northern China

**DOI:** 10.1038/srep34184

**Published:** 2016-09-29

**Authors:** Fujun Niu, Guodong Cheng, Yonghong Niu, Mingyi Zhang, Jing Luo, Zhanju Lin

**Affiliations:** 1 State Key Laboratory of Frozen Soil Engineering, Cold and Arid Regions Environmental and Engineering Research Institute, Chinese Academy of Sciences, Lanzhou 730000, China

## Abstract

Permafrost is determined to a large extent by the Earth’s surface temperature, therefore it distributes mainly in high altitude and latitude regions. However, stable, warm (about −1 °C) permafrost occurs within a scree slope in northern China that is more than 600 km south of the southernmost limit of latitudinal permafrost on the Eurasian Continent. It is at an elevation of only 900 m above sea level (ASL). The area has a mean annual air temperature (MAAT) of 6 to 8 °C. Thermal processes of the scree slope, investigated through field monitoring and numerical simulation, showed that the permafrost is caused by winter air convection within the porous rock deposits and is stable as air convection does not occur in summer time. The deposit is covered by a 30-cm-thick peaty soil layer dated (carbon C-14) to between 1,000 to 1,600 years ago. The layer also contributes to the permafrost’s existence due to the peat’s thermal conductivity offset when frozen and thawed. The existence of permafrost under such warm climatic conditions confirms the effectiveness of using crushed rock layer as basement or slope cover to protect the warm permafrost subgrade of the recently-constructed Qinghai-Tibet Railway (QTR), even under the predicted climate warming conditions.

Permafrost is defined as ground (soil or rock and including ice or organic material) that remains at or below 0 °C for at least two consecutive years. Permafrost underlies about 25% of the earth terrestrial surface^1^. In the northern hemisphere, it occupies extensive areas in the high latitudes of North America and Eurasia as well as the high-elevation plateau terrains of the mid- and low-latitudes. Permafrost also occurs at high elevation in many mid-latitude mountains (Fig. 1). Climate warming trends in the last three decades, many observations and monitoring have confirmed the extensive degradation of permafrost^2^,^3^,^4^.

However, cases of permafrost occurrence under scree or talus slopes far from the common permafrost regions have not often been reported in the Northern Hemisphere (Fig. 1). Permafrost distribution on a talus slope in the Alps was the first studied case in Europe^5^. After that, many cases were studied in Europe, concentrated in the Alps area^6^,^7^,^8^,^9^,^10^,^11^ and in Norway^12^. It was also discovered in Plateau Mountain in South-west Alberta in North America^13^. In central and high Asia, three cases were studied on the Qinghai-Tibet Plateau^14^ and in the Tianshan Moutains^15^. Such phenomena are also found in Hokkaido, northern Japan^16^. The low ground temperature phenomenon (LGTA), observed in these areas, can be explained as the result of the Horton-Rogers-Lapwood convection (HRLC) after Rayleigh-Bénard convection (RBC)^17^. Such explanations were confirmed by natural phenomena, experiments and numerical simulations^18^,^19^. Among the reported cases, the one in Pingquan County of Hebei Province, Northeast China with the ground temperature significantly lower than the adjacent soil or bedrock, is the southernmost case in northern hemisphere.

In Northern China, local residents were surprised, in 2011, by finding cold earth on a mountain scree slope in hot (up to +30 °C plus) summers. Then the existence of a shallow body of perennially-frozen ground and its ice were investigated systematically. The site (marked as L-15 in Fig. 1) is located in a mountain scree slope located in Pingquan County, Hebei Province (41°13′10″N, 118°51′26″E; 900 m above sea level, ASL.). The area experiences mean annual air temperature (MAAT) between 6 and 8 °C with mean January and July air temperatures of −25 °C and 33 °C, respectively. Moreover, in July 2008, soils with consistent subzero temperatures persisted following a forest fire on the slope. This permafrost phenomenon is unusual because the site is 600 km south of the southernmost limit of permafrost on the Eurasian Continent, and 600 km from the alpine permafrost in the Da Xing’anling Mountains of Northeast China. The research focused on the cooling mechanism enabling the permafrost’s occurrence under the scree slope, and increasing the possibility of cooling roadbed in permafrost regions.

## Results

**Figure 2:**
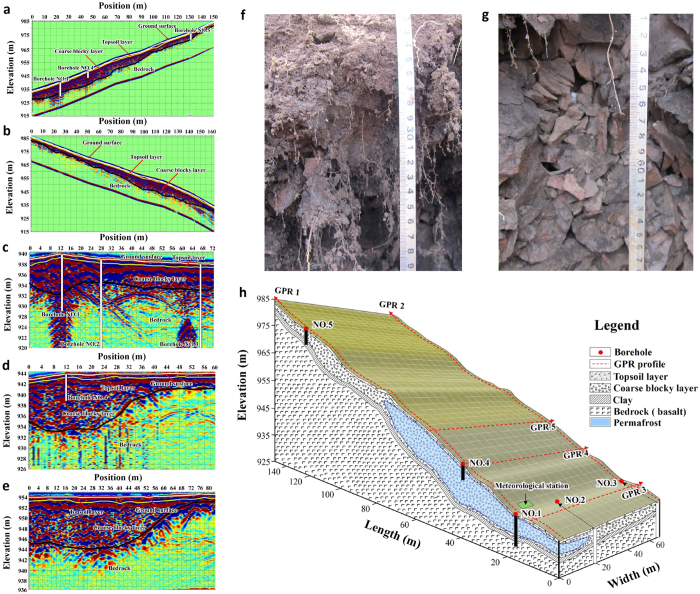
The stratigraphy of the permafrost location as revealed by borehole and GPR data. (**a~e**) GPR exploring profile #1~#5, showing the coarse block distribution in the slope. (**f **) Surface peat layer with a thickness around 30 cm. This layer provides a property same as a kind of thermal semi-conductor around a year, for the tested thermal conductivity in wither time is 1–2 times of that in summer time. (**e**) The porous coarse block layer with an maximum thickness of 11 m, and the block size ranges mainly within 5–15 cm. (**h**) The geological sketch map of the monitored slope, and also the frozen body is zoned with blue color.

The site was investigated by borehole drilling and ground penetrating radar (GPR) in 2011 (Fig. 2). Five boreholes (No. 1–5) were drilled within the slope and used to construct the site stratigraphy. Thermistors, installed within the boreholes, monitored ground temperatures between the ground surface and a depth of 11 m. The GPR soundings (GPR #1 to GPR #5), shown as Fig. 2a–e, show a layer of peaty soil (Fig. 2f), usually around 30 cm thick which overlies a coarse block layer (Fig. 2g) with varying thickness. This is well illustrated in boreholes from No. 1 to No. 5 and the GPR soundings, indicating that the blocky layer has a maximum thickness up to 11 m. The blocky layer is underlain by basalt bedrock.

**Figure 3:**
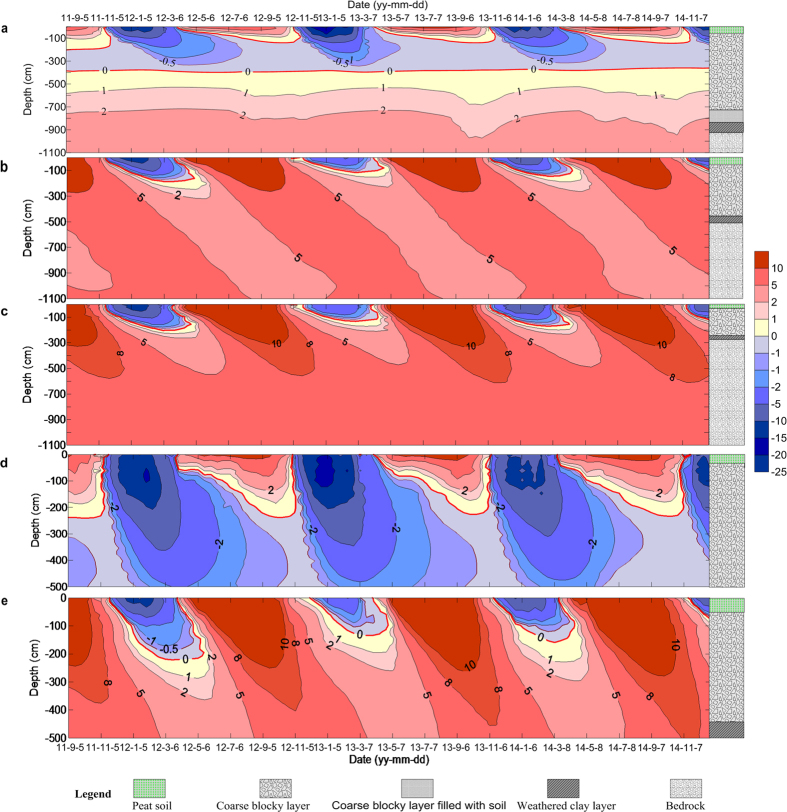
Spatial and temporal distribution of ground temperatures in the 5 monitoring boreholes on the ‘cold’ scree slope (unit: °C). (**a**) Ground temperature in borehole No. 1, showing a permanent frozen zone from 2 m to 4 m in depth. (**b,c,e**) Ground temperature in boreholes No. 2, No. 3 and No. 5, showing a existence of 2-m thick seasonal frozen ground. (**d**) Ground temperature in borehole No. 4, showing a permanent frozen zone from 2 m to more than 5 m in depth.

**Figure 4:**
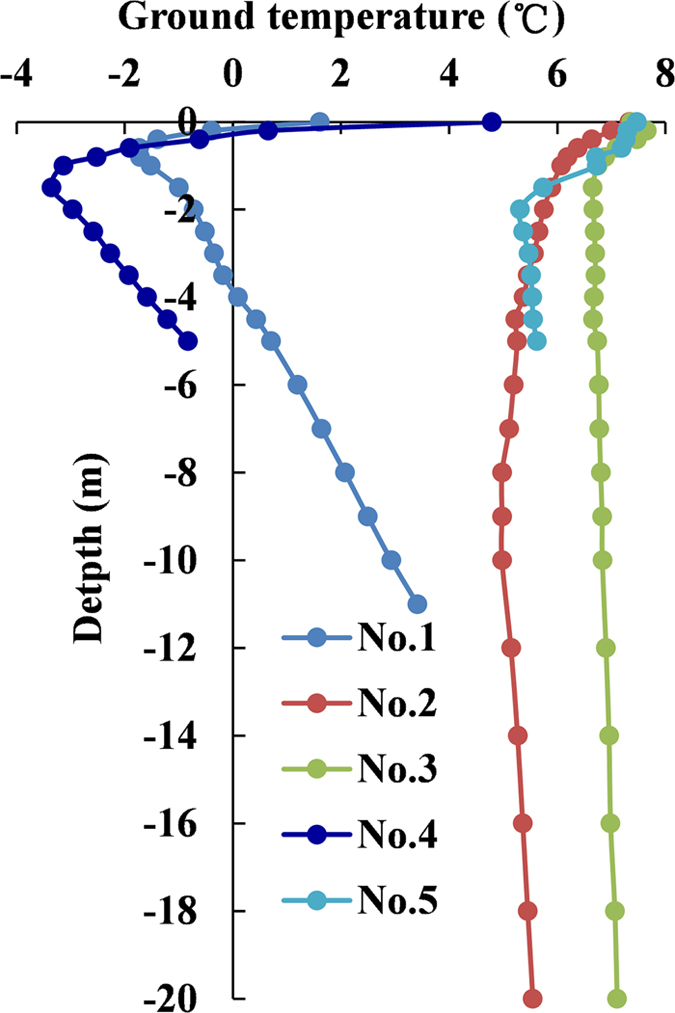
Average ground temperature profiles in the 5 monitoring boreholes from 2011 to 2014. Temperature profiles show that the ground temperatures with negative values in boreholes No. 1 and No. 4 were wholly lower than that in boreholes No. 2, No. 3 and No. 5.

Figure 3 shows temporal and spatial distributions of the ground temperatures measured in the five boreholes. The ground temperatures between 1.5 m to 4 m in borehole No. 1 were −4.5 °C to 0 °C during the monitoring period (Fig. 3a), which was more than 3 years. While in borehole No. 4, temperatures ranged from −8.1 °C to 0 °C at depths between 2.5 m to 5.0 m (Fig. 3d). Figure 4 shows the averaged ground temperature profiles in the five boreholes within the period from 2011 to 2014. The lowest averaged ground temperature at a depth of 0.8 m and 1.5 m in borehole No. 1 and No. 4 was −1.7 °C and −3.4 °C, respectively. While the averaged ground temperatures at every monitored depths in the other borehole were not lower than 5 °C. Therefore, permafrost exists in the slope in an area where the MAAT is 6 to 8 °C, detected from the borehole No. 1 and No. 4. There were no frozen layers in summer in boreholes No. 2, No. 3 and No. 5 (Fig. 3b,c,e). Accordingly, the ground temperatures monitored in the five boreholes show differences that reflect the varied thicknesses of the coarse blocky layer. For example, in borehole No. 1, the coarse blocky layer is 8.4 m in thickness and the permafrost thickness of 2.5 m was measured. In comparison, at borehole No. 4, the 11 m thick coarse blocky layer has permafrost thicker than 2.5 m.

It can be also seen from Fig. 3 that temperature within the coarse blocky layer is very sensitive to external (surface) temperatures during the freezing period. When air temperature drops sharply in mid-November, the cold, dense air penetrates rapidly into the voids between the blocks, resulting in a near-heterogeneous temperature distribution in the blocky layer from top to bottom due to air convection within the porous material. During the early thaw period, the isotherms are laterally parallel indicating that the air in the voids is stable and there is no convective heat transfer between the warmer (lighter) air above and the colder (denser) air below. Such a thermal regime indicates the heat exchange between the coarse blocky layer and the ambient environment is relatively instantaneous and intense in winter, while prolonged and feeble in summer. In summary, the permafrost results from the seasonal thermal diode. The major differences between boreholes No. 1 and No. 4, and boreholes No. 2, No. 3 and No. 5 is the thickness of the coarse blocky layer, and fine particles filling in the pores, thus resulting in the observed ground temperature deference. According to drilling and GPR investigations, the thickness in boreholes No. 1 and No. 4 is over 8.0 m, while it is less than 4.0 m in the rest boreholes. In areas where the coarse blocky layer is either thinner than 1.5 m or absent, the ground thermal regime is characteristic of seasonally-frozen ground.

## Discussion

The dominant controlling factor over ground freezing at the site is the HRLC effect induced by the closed coarse block layer on the slope. Because cold air is denser than warm air, it tends to displace warmer air in the pore spaces between the blocks and causes air convection in winter time; while in summer time, no convective heat transfer takes place and heat exchange is mainly in the form of conduction. Essentially, the imbalance of heat losses in winter and heat absorbs in summer produces a net heat loss around a year and thus lowers the ground temperature.

The ground thermal data at the site suggests that the near-surface peaty soil layer serves as a kind of thermal diode. Because site precipitation is concentrated in the summer months, this results in a high moisture content of the surface peaty soil prior to winter freezing. It is well known that the thermal conductivity of icy peaty soil increases by about 1–2 times over dry and unfrozen peat which, in the summer months, serves as an excellent thermal insulator^20^,^21^. Therefore, in a year, the peat layer has the thermal properties of a thermal diode or semi-conductor.

**Figure 5:**
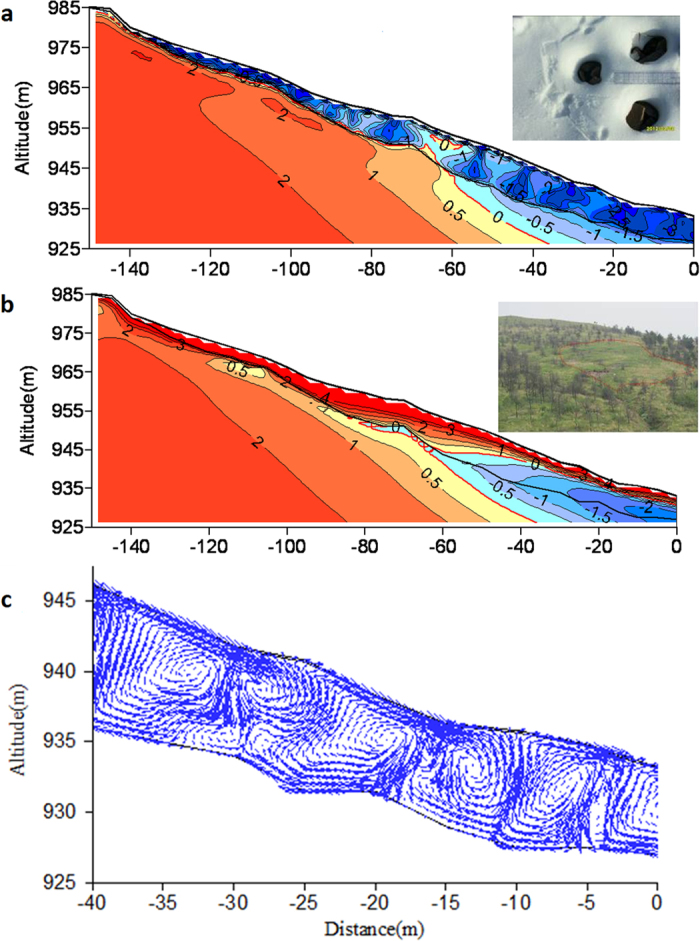
Simulated ground temperature fields (Unit: °C) in summer and winter and air flow field in the ‘cold’ scree slope. (**a**) Circular isotherms in the blocky layer in February and holes in the heat vent from hoar developing snow cover indicate heat exchange is controlled by air convection in winter, the photograph was taken on March 9, 2012. (**b**) Parallel isotherms in August indicate that heat exchange is dominated by conduction in summer, and the photograph taken on August 6, 2011 shows that trees are sparse in the low ground temperature region enclosed by red boundary. (**c**) The simulated air flow field in the vertical profile of the main part of the blocky slope in winter.

To further investigate the heat exchange process, the site was modeled with a numerical simulation using the field monitored boundary conditions and measured material parameters to define the processes. Figure 5 shows the heat exchange was greatest in the blocky layer in winter; as indicated by the circular pattern of isotherms and air flow circles on February 1^st^ (Fig. 5a,c) and the parallel isotherms on August 1^st^ (Fig. 5b). The heat flux in the main part of the blocky layer was around 6 W/m^2^ in winter and 4 W/m^2^ in summer. This difference was reflected on the slope by holes (Fig. 5a) in the snow cover in winter and the sparse vegetation in the low ground temperature region in summer (Fig. 5b).

The surface peaty soil was AMS-^14^C dated at 1,040 years B. P. (top) and 1,590 years B. P. (base, at depth of 35 cm) by Bata Analytic Company in USA (sample numbers Beta −352496, 352496, and error is +/−30 a). Therefore, the blocky scree is around 1,600 years old at least. Combining the data from field monitoring and laboratory modeling, the cooling effect of the blocky layer might have persisted during the climate warming periods of the Holocene Megathermal, medieval and latest decades. Whether ground-ice was developed or not in the shallow part was related to the local climate, since the area was involved in the global semi-arid climate changing process in last 60 years^22^,^23^. The frozen ground also survived a forest fire in 2008. Taking into account the local MAAT of approximately 6.3 to 7.8 °C and the mean annual ground surface temperature (MAGST) of approximately 5.3 to 6.8 °C, the blocky permafrost layer will probably survive through a predicted climate warming of approximately 2.3 to 4.2 °C in the 21^st^ century.

Such naturally occurring ‘cold spot’ phenomena have practical implications for engineering design and construction in cold regions. In China, Cheng proposed a principle of ‘proactive cooling’ for designing and building roadbeds above ice-rich permafrost^24^. In engineering applications, crushed rock layers can effectively protect underlying warm and ice-rich permafrost. This is a technique that has been used for the Qinghai-Tibet Railway (QTR). Monitoring data for 10 years shows that such a roadbed structure is successful in cooling the foundation materials in both the active layer and the underlying permafrost, as indicated by the aggrading permafrost table (0 °C isotherm) and expanding of the −1 °C range under the embankment^25^. Such a changing trend of the ground temperature guarantees the thermal stability of QTR roadbed, even under a climate warming of 2.2 °C to 2.6 °C in 50 years on the Qinghai-Tibet Plateau^26^.

## Methods

### Site description and monitoring measurements

Peat soil and rock samples for this study were collected at the described site (41°13′10″N, 118°51′26″E), located in Pingquan County, Hebei Province, China, with a distance of around 300 km from Beijing. The ground temperatures were measured with thermistors, with a precision of ±0.02 °C, manufactured and assembled by the State Key Laboratory of Frozen Soil Engineering. The air temperature data of the Pingquan County was collected from the Pingquan County Weather Station, and the air temperature on the studied slope was collected by a simple weather station installed in 2011. The ground temperature data were collected automatically by a CR3000 data logger four times per day.

### Numerical simulation

An air convective heat transfer model with non-Darcy flow for porous media material was used to simulate the cooling process and effect of the natural rock layer located at the site. Temperature and velocity fields were obtained based on the *in-situ* thermal boundaries and geo-material characteristics. The temperature distributions were in accordance with the observed results, suggesting that the simulated results were reasonable and valuable.

## Additional Information

**How to cite this article**: Niu, F. *et al*. A naturally-occurring ‘cold earth’ spot in Northern China. *Sci. Rep.* **6**, 34184; doi: 10.1038/srep34184 (2016).

## Figures and Tables

**Figure 1:**
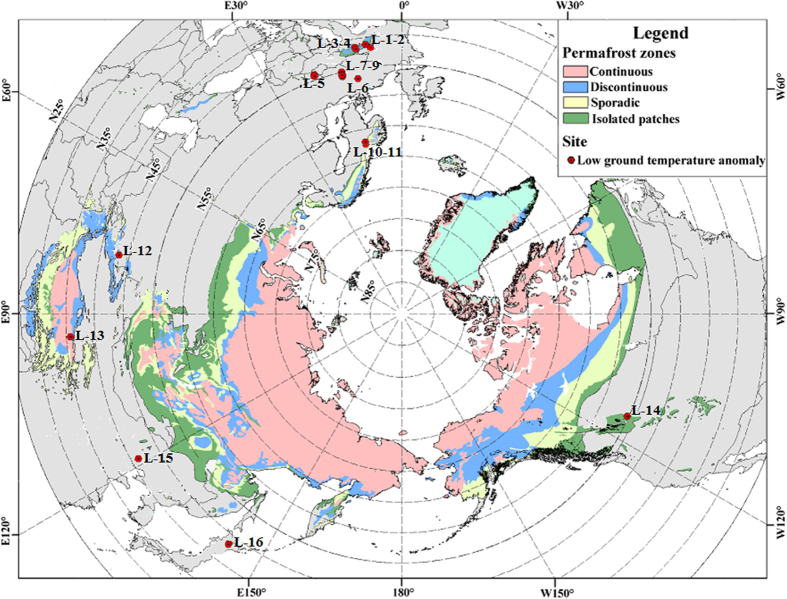
Distribution of low ground temperature anomalies outside of the continental permafrost bodies. Maps were generated using GIS software (ArcGIS 8.0, http://resources.arcgis.com/), and the permafrost distribution data was from the International Permafrost Association^27^. Many ‘cold earth’, i.e. low ground temperature anomaly (LGTA, marked as L-number for short) have been reported to occur on scree or talus slopes spreading away from the present southern or lower limit of permafrost (Table 1). The studied robust permafrost site (marked as L-15) in North China is the southernmost in the Northern Hemisphere, except the one marked L-13, which is on the Qinghai-Tibet Plateau with much higher elevation (4,700 m ASL.).

**Table 1:** Typical cold earth spot distribution in the North Hemisphere.

Site	Latitude	Longitude	Elevation (m; a.s.l.)	MAAT (°C)	MAGT (°C at a depth)	Reference
L1: Mattertal, Valais, Swiss Alps	46°11′N	7°51′E	2,600–2,900	−0.7*	−2.3 ∼−2.2 °C (1.8∼1.5 m)	5
L2: Creux du Van, Switzerland	46°56′N	6°44′E	1,170–1,300	5.5	0.5∼2.0 (0.3∼0.7 m)	6
L3: Upper Engadin, Swiss Alps	46°30′N	9°49′E	2,200–2,900	−1.8	−0.5∼2.3 (>4 m)	7
L4: Flüela Pass, Swiss Alps	46°44′N	9°56′E	2,380–2,600	0.2	−1.3∼0 (6 m)	8
L5: Tatra Mountains, Poland and Slovakia	49°7′–49°17′N	19°32′–20°19′E	1,450–2,000	−4∼6	−0.8∼1.8 (0.3 m)	9
L6: Odertal, Harz Mountains, Central Germany	51°44′N	10°33′E	600	6.2	1.6 (0.3∼1 m)	10
L7: Klic hill, Central Europe	50°47′N	14°34′E	542	6.8	−0.1 (0.5∼0.7 m)	11
L8: Kamenna Hura, Central Europe	50°42′N	14°21′E	300	7.5	−1.6 (0.5∼0.7 m)	11
L9: Klic hill, Central Europe	50°49′N	14°04E	580	7.5	0.1 (0.3∼1 m)	10
L10: Mountains Elgåhogna, Norway	62°09′N	11°57′E	1,295–1,335	—	1.3∼2.0 (1 m**)	12
L11: Mountains Sølen, Norway	61°55′N	11°31′E	1,440–1,420	—	2.6∼4.2 °C (1 m**)	12
L12: Transili Alatau Range, Kazakhstan	43°03′N	77°15′E	2,550	1.4	−1.2 ∼ −2.7 (3.5∼6.0 m)	15
L13: Kunlun Shan, Qinghai, China	35°41′N	94°02′E	4,820	−6	−2.6 (0.9 m)	14
L14: Plateau Mountain, Alberta, Canada	50°11′N	114°30′W	1,950	−1.17	4 °C (0.7 m***)	13
L15: Pinquan, Hebei, China	41°13′N	118°51′E	900	6∼8	−1∼0 (2 m)	This study
L16: Nishi-Nupu-kaushinupuri Mountain, Northern Japan	43°16′N	143°05′E	1,251	1.3∼1.7	—	16

^*^Ground surface temperature.

^**^Lower than in nearby till and bedrock.

^***^Lower than in nearby mineral soils.
